# Cervical Spine Magnetic Resonance Imaging Findings in Hirayama Disease

**DOI:** 10.7759/cureus.40015

**Published:** 2023-06-05

**Authors:** Tushar Kalekar, Aparna S Prabhu, Suhas M

**Affiliations:** 1 Radiodiagnosis, Dr. D. Y. Patil Medical College, Hospital & Research Centre, Dr. D. Y. Patil Vidyapeeth, Pune, IND

**Keywords:** neurology, neuroradiology, mri, cervical flexion myelopathy, hirayama disease

## Abstract

Background

Hirayama disease is an uncommon type of cervical cord myelopathy seen typically in young males due to trauma from flexion movements. This study aims to assess the clinical presentations and classify the extent of various cervical spine MRI findings for the local population.

Methodology

A retrospective study of 13 patients diagnosed with Hirayama disease on cervical MRI was performed from January 2017 to December 2022 at Dr. D. Y. Patil Medical College, Hospital and Research Center, Pune.

Results

Of the 13 patients, 12 (92%) were male, and one (8%) was female. Nine (69%) patients were in the 16-25-year age group, two (15%) were in the 26-35-year age group, and one (8%) each was in the 6-15-year and 66-75-year age groups. Upper limb weakness was the most common clinical symptom seen in 12 (92%) patients, followed by distal muscle atrophy in seven (54%) patients. Tremors in the hand were a rare symptom seen in two patients. Claw hand was an atypical symptom seen in one patient. On cervical MRI, all patients showed excessive forward shifting of the posterior dura on flexion, with resultant cord compression due to tightness of the dural sac. One (8%) patient had no signs of myelopathy, while 12 (92%) patients had developed chronic myelomalacia and showed abnormal cord hyperintensity and atrophy in the lower cervical cord. All 13 (100%) patients showed increased laminodural space on flexion; the mean thickness was 4.08 mm, with the minimum and maximum thickness being 2.4 mm and 6.7 mm, respectively. Classifying by length of the anterior bulging dura, one (8%) patient showed involvement of less than two vertebral body segments, eight (62%) patients showed involvement of two to four vertebral body segments, and four (30%) patients showed involvement of more than four vertebral body segments. Crescent-shaped post-contrast enhancement on flexion was seen in all eight (100%) patients who underwent a contrast study. Prominent epidural flow voids on flexion were seen in six (46%) patients.

Conclusions

Hirayama disease is an uncommon type of cervical myelopathy seen typically in juvenile males. The occult onset of distal upper limb weakness and atrophy during puberty, typical MRI features of lower cervical cord atrophy, and the presence of a crescent-shaped enhancing mass in the posterior epidural space are pathognomonic of the condition. A few atypical cases can also occur. Early diagnosis and treatment are crucial to avoiding serious dysfunction.

## Introduction

Hirayama disease is a rare type of cervical myelopathy caused by trauma from flexion movements of the neck. It typically occurs in young Asian males in their second or third decade [1,2]. The underlying etiology is considered to be necrosis of anterior horn cells due to chronic microcirculatory changes in anterior spinal artery territory induced by repeated or sustained neck flexion [3]. It typically presents with unilateral weakness and amyotrophy of the distal upper limb in the hand and forearm. It may also present with asymmetric bilateral upper limb involvement, and symmetric involvement is rare. There is a sparing of sensory involvement, and occasionally coarse tremors can occur [3,4]. The disease is considered to progress for three to five years and then have a stationary phase [2].

On MRI, in a neutral position, intramedullary high signal intensity on T2 weighting and subtle asymmetric lower cervical cord atrophy can be seen. However, MRI with cervical flexion is suggested and shows forward displacement of the posterior dura, increased laminodural space, and post-contrast enhancement of a crescent-shaped posterior dural sac. Prominent epidural flow voids may also be seen on flexion [2-4].

In this study, we aim to assess the prevalence and clinical presentations of Hirayama disease in the local population. Emphasis has been placed on unusual cases to add to the relevant literature. We assess the imaging findings to ascertain the most common and objective findings for diagnosis. Furthermore, we classify these changes based on the extent of involvement.

## Materials and methods

Study design

A retrospective, cross-sectional study was conducted among patients who were diagnosed to have Hirayama disease on cervical MRI performed over a period of six years from January 2017 to December 2022 at Dr. D.Y. Patil Medical College, Hospital & Research Center, Pune.

Inclusion and exclusion criteria

Patients of all age groups who had a principal diagnosis of Hirayama disease on MRI were included. Patients who had not undergone MRI with cervical flexion or had inconclusive imaging findings for Hirayama disease were excluded.

A total of 13 patients with Hirayama disease were found on a review of the hospital picture archiving and communication system (PACS) and were included in the study.

Standard cervical spine protocol (sagittal T2 tse, sagittal T1 tse, coronal T2 stir, axial T2 tse, axial T1 tse) with additional sagittal T2 tse and axial T2 tse on cervical flexion were obtained for all patients. Eight of the 13 patients had undergone a contrast study, and post-contrast T1 sagittal and T1 axial images were available.

A waiver letter was obtained from the Institutional Ethics Sub-Committee, Dr. D. Y. Patil Medical College, Hospital & Research Centre, Pune (approval number: I.E.S.C./03/2023 dated 18/03/2023).

Data collection

Clinical information including patient age, clinical symptoms, and MRI images and reports were accessed from the hospital PACS. 

Statistical analysis

Categorical variables for sex, age group, clinical, and MRI findings have been expressed as numbers and percentages, and the results are presented in tables.

## Results

Table 1 shows the sex distribution of the patients. Among the 13 patients studied, 12 were male and only one was female.

**Table 1:** Sex distribution.

Sex	Number	Percentage
Male	12	92%
Female	1	8%
Total	13	100%

Table 2 shows the sex distribution of patients. The majority of the patients were young, with nine patients aged between 16 and 25 years and two aged between 26 and 35 years. The youngest was a girl aged seven years, and the oldest was a 75-year-old male.

**Table 2:** Age distribution.

Age group	Number	Percentage
6–15	1	8%
16–25	9	69%
26–35	2	15%
36–45	0	0
46–55	0	0
56–65	0	0
66–75	1	8%
Total	13	100%

Table 3 summarizes the presenting clinical symptoms of patients. Upper limb weakness was the most common symptom seen in 12 (92%) patients. It was unilateral in seven and bilaterally asymmetric in five patients. Upper limb muscle atrophy was the second most common symptom seen in seven (54%) patients. It was unilateral in four (31%) and bilateral in three (23%) patients. One patient each presented with unilateral and bilateral upper limb tremors, which was a relatively rare symptom. A seven-year-old female child presented with the atypical symptom of bilateral claw hands.

**Table 3:** Classification based on clinical presentation.

Clinical symptoms	Site	Frequency	Percentage
Upper limb weakness	Unilateral	7	54%
	Bilateral	5	38%
Upper limb muscle atrophy	Unilateral	4	31%
	Bilateral	3	23%
Upper limb pain	Unilateral	1	8%
	Bilateral	0	0
Upper limb tremors	Unilateral	1	8%
	Bilateral	1	8%
Atypical: claw hand	Bilateral	1	8%

Table 4 summarizes the MRI findings seen in patients. All patients showed excessive forward shifting of the posterior dura on flexion, with resultant cord compression due to tightness of the dural sac. One patient showed only mild cord compression without signs of myelopathy, while 12 patients showed cervical cord compression with an abnormal T2 hyperintense cord signal and asymmetric cord atrophy. The levels at which patients showed forward shift of the posterior dura and cord compression have been summarized in the table. All 13 patients showed increased laminodural space on flexion. The maximum laminodural distance (anteroposterior distance) seen in patients on flexion and the length (craniocaudal distance) of increased laminodural space is also summarized in the table. Prominent epidural flow voids on flexion were seen in six (46%) patients. All eight patients who underwent the contrast study showed enhancement of the epidural component on flexion. A posterior disc bulge with nerve root compression was an incidental age-related finding seen in the elderly patient and was not related to Hirayama disease.

**Table 4:** MRI findings with classification based on the extent of involvement.

MRI findings	Extent of involvement	Frequency	Percentage
Cord compression without abnormal cord signal	-	1	8%
Cord compression with abnormal cord signal	-	12	92%
Cord compression with asymmetric cord atrophy	-	12	92%
Level of cord compression	C3–C5	1	8%
	C4–C6	3	23%
	C4–C7	1	8%
	C5–C6	4	31%
	C5–C7	4	31%
Increased laminodural space on flexion	-	13	100%
Maximum laminodural space on flexion	2.1–3 mm	3	23%
	3.1–5 mm	7	54%
	5.1–7 mm	3	23%
Segment of cord showing increased laminodural space	<2 vertebral body segments	1	8%
	2–4 vertebral body segments	8	62%
	>4 vertebral body segments	4	30%
Prominent epidural flow voids on flexion	-	6	46%
Enhancement of epidural component on flexion in patients undergoing contrast study	-	8	100%

**Figure 1:**
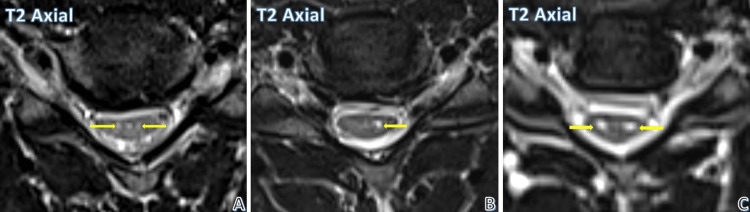
Types of intramedullary cord hyperintensities. Subtle T2 intramedullary cord hyperintensities (A), unilateral hyperintensity (B), and bilateral hyperintensities (C) (yellow arrows depict cord hyperintensities in all images).

Figure 1 depicts the intramedullary high signal intensity seen on T2-weighted images. Few patients had only subtle linear hyperintensity, as seen on the axial T2-weighted image in Figure 1A. Other patients showed high signal intensity in the region of the anterior horn cell in axial T2-weighted images, which was either unilateral (as in Figure 1B) or bilateral (as in Figure 1C).

**Figure 2:**
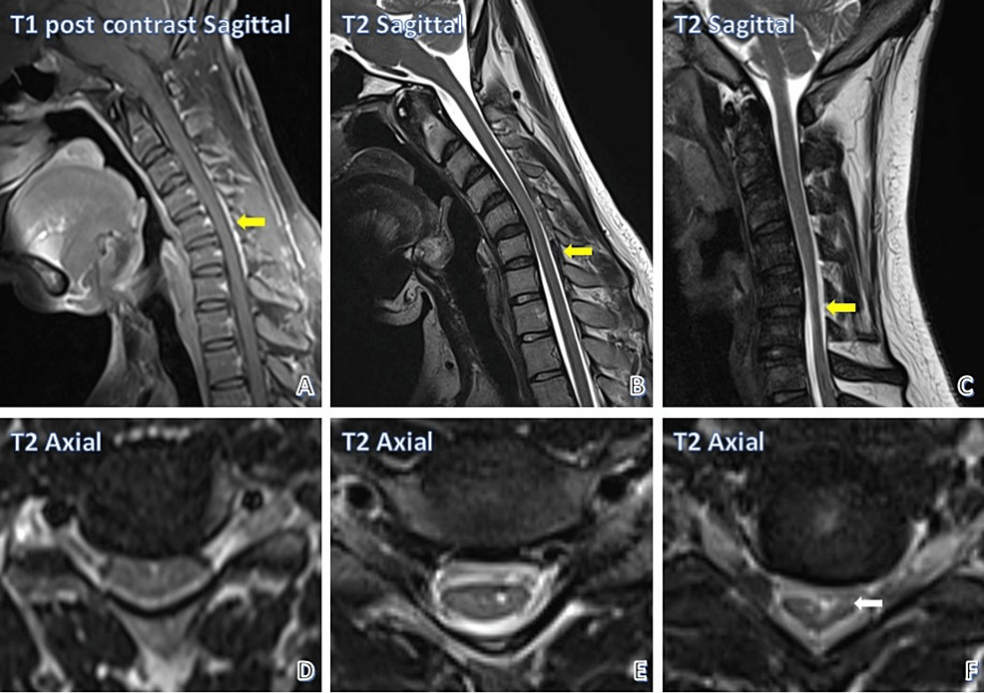
Cord compression and various stages of myelomalacia. Cord compression without abnormal cord signal intensity in a patient (A, D). Cord compression with abnormal cord intensity in a patient (B, E). Cord compression with atrophy (white arrow) in a patient (C, F) (yellow arrows show the level of compression in all sagittal images).

Figure 2 depicts cord compression and various stages of myelomalacia. Figure 2A and Figure 2D show a patient with cord compression without an abnormal cord signal; Figure 2B and Figure 2E show a patient with cord compression with an abnormal cord signal; and Figure 2C and Figure 2F show a patient with cord compression causing cord atrophy.

**Figure 3:**
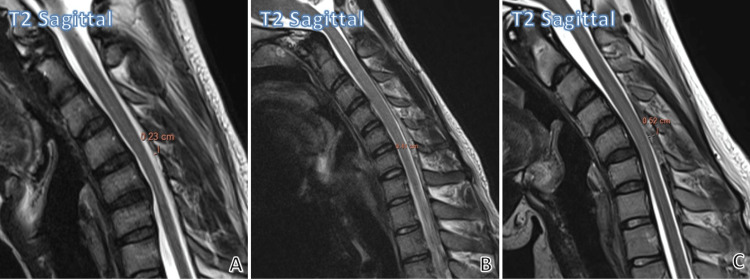
Different extent of the maximum anteroposterior thickness of the laminodural space on flexion. Mild increase in laminodural space thickness (A), moderate increase in laminodural space thickness (B), and severe increase in laminodural space thickness (C) (maximum anteroposterior thickness of the laminodural space on flexion are shown in mid-sagittal images).

Figure 3 depicts the different extents of the maximum anteroposterior thickness of the laminodural space on flexion.

**Figure 4:**
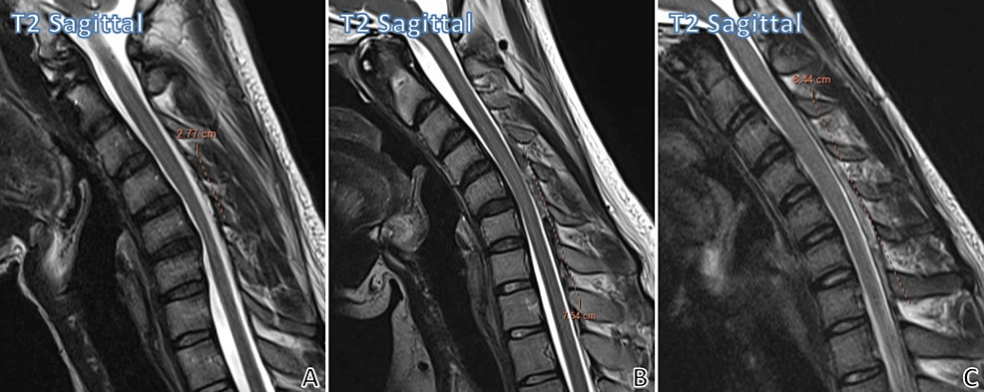
Different craniocaudal extent of increased laminodural space on flexion. Mild craniocaudal extent of increased laminodural space (A), moderate craniocaudal extent of increased laminodural space (B), and severe craniocaudal extent of increased laminodural space (C) (craniocaudal lengths of increased laminodural space on flexion shown in each mid-sagittal image).

Figure 4 depicts the different craniocaudal extents of increased laminodural space on flexion.

**Figure 5:**
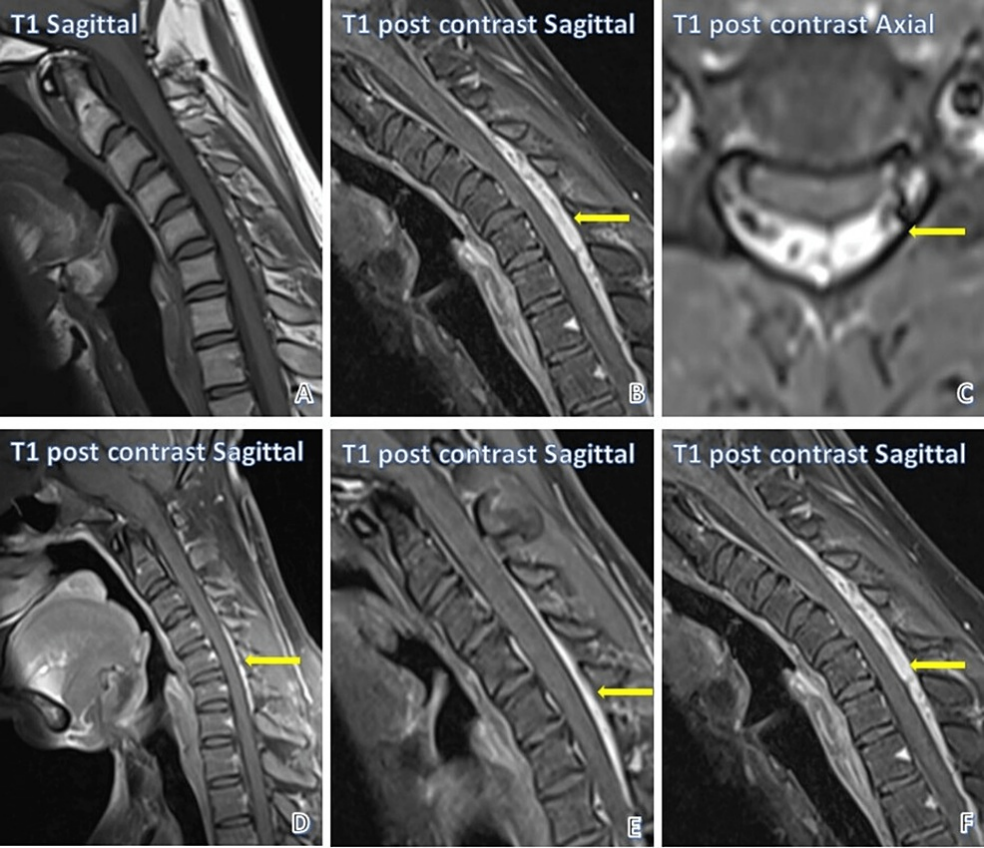
Contrast enhancement of the posterior epidural space. A patient with increased laminodural space on the flexion T1 sagittal image (A), showing post-contrast enhancement on sagittal (B) and axial (C) images. Contrast enhancement is seen in a case with- a mild increase in laminodural space (D), a moderate increase in laminodural space (E), and a severe increase in laminodural space (F) (yellow arrows depict contrast enhancement of the posterior epidural space in all images).

Figure 5 depicts the contrast enhancement of the posterior epidural space seen in the post-contrast T1-weighted image with flexion of the neck. Figure 5A shows a sagittal pre-contrast T1-weighted image with flexion, and Figure 5B and Figure 5C show a post-contrast T1-weighted image with flexion in the same patient. Figures 5D-5F depict sagittal post-contrast T1-weighted images in different patients, demonstrating the different extent of involvement.

## Discussion

Hirayama disease was first described by Japanese neurologist Keizo Hirayama in 1959 [5]. The entity is also called juvenile muscular atrophy of the distal upper extremities. Hirayama disease is a male-prone disease occurring most commonly between 15 and 25 years. It is characterized by unilateral or asymmetric bilateral weakness of the hand and forearm supplied by C7-T1 myotomes and sparing of the brachioradialis muscles. There is no lower limb involvement or sensory disturbance. Although the pathogenesis is unknown, the currently accepted theory is the disproportionate growth between the length of the spinal cord and the length of the spinal column during growth spurt which leads to excessive anterior bulging of the posterior dura during neck flexion. Long-term compression of the cervical cord due to the tightness of the dural sac during flexion leads to microcirculatory changes in the region supplied by the anterior spinal artery, causing ischemia and necrosis of anterior horn cells [2].

Our study shows Hirayama disease has a male preponderance, with 16-25 years being the most affected age group. Among the two atypical presentations, one was a seven-year-old female with a claw hand, asymmetric upper limb weakness, and muscle atrophy. Another was a 75-year-old male patient with unilateral upper limb weakness. Based on the imaging findings which showed asymmetric lower cord atrophy, intramedullary hyperintensity on T2 weighting, and prominence of the posterior epidural space on flexion images, a diagnosis of Hirayama disease was suggested. Upper limb weakness was the most common clinical symptom seen in 12 (92%) patients, being unilateral in seven (54%), and asymmetric bilateral in five (38%). Only one (8%) patient did not complain of weakness but had unilateral upper limb pain and tremors instead. Distal muscle atrophy was the second most common symptom seen in seven (54%) patients; four had unilateral and three had bilateral weakness. Tremors in the hand were a rare symptom seen unilaterally and bilaterally in one patient each.

On MRI in a neutral position, the condition is suspected if there is asymmetric cord flattening and atrophy of the lower cervical cord. The presence of intramedullary high signal intensity on T2 weighting is also a feature [1]. An MRI with neck flexion is recommended in suspected cases [4]. There is a characteristic forward shifting of the posterior dura during neck flexion [6]. Most patients with Hirayama disease have a crescent-shaped loss of attachment behind the posterior dura, which may show post-contrast enhancement [7]. Prominent epidural flow voids may also be seen on flexion [2-4].

Among our 13 cases, 12 (92%) showed the presence of high signal intensity on T2-weighted images and asymmetric cervical cord atrophy on neutral MRI. Only one patient showed cord compression and had not yet developed abnormal cord intensity or cord atrophy. This demonstrates that most cases have progressed to the stage of chronic myelomalacia at the time of initial presentation. A high degree of awareness and suspicion should be exercised to diagnose Hirayama disease at an early stage. Abnormal cord intensities were seen bilaterally in six (50%) patients, unilaterally in one (8%) patient, and subtly in five (42%) patients. Abnormal forward shift of posterior dura and cord compression was seen at the C3-C5 level in one patient, C4-C6 in three patients, C4-C7 in one patient, C5-C6 in four patients, and C5-C7 in four patients. On MRI with flexion, all 13 patients showed increased laminodural space. This suggests that increased laminodural distance on flexion images is one of the best imaging features for diagnosing Hirayama disease. We have further classified the extent of increased laminodural space based on the maximum anteroposterior thickness and length of involvement. On flexion MRI, three (23%) patients showed thicknesses of 2.1-3 mm, seven (54%) patients showed thicknesses of 3.1-5 mm, and three (23%) patients showed thicknesses of 5.1-7 mm. The mean thickness was 4.08 mm, with the minimum and maximum thicknesses being 2.4 mm and 6.7 mm, respectively. Next, classifying by length of anterior bulging dura, one (8%) patient showed involvement of fewer than two vertebral body segments, eight (62%) patients showed involvement of two to four vertebral body segments, and four (30%) patients showed involvement of more than vertebral body segments. Crescent-shaped post-contrast enhancement on flexion was seen in all eight patients who underwent the contrast study. Prominent epidural flow voids on flexion were seen in six (46%) patients on flexion. A posterior disc bulge with nerve root compression was a rare feature seen only in our one elderly patient. This is a coincidental age-related finding not related to Hirayama disease.

Although Hirayama disease is considered a benign condition with a stationary course, studies have shown that it can cause serious dysfunction if not treated early. Therefore, restriction of neck flexion conventionally by neck collar support or by a surgical method is the main treatment for Hirayama disease [8-14].

Tashiro et al. conducted a nationwide epidemiological study of patients with Hirayama disease in Japan, which has information on the number of patients affected per year, distribution of age of onset, time between onset and quiescence, neurological symptoms and signs, and effects of conservative treatment. There was a marked male preponderance, the peak age group affected was 15-17 years and showed slow onset and progression, predominantly involving unilateral forearm and hand with cold paresis, and quiescence by six or fewer years [15].

In a study by Huang et al., a review of 40 patients with Hirayama disease was done in Taiwan. Overall, 87.5% of the patients were male, and the mean age of onset was 16.8 years. Progressive distal muscle weakness and atrophy were seen predominantly in the right upper limb, with cessation of progression within five years. About one-third of patients had a history of heavy physical activity before the development of symptoms. MRI showed anterior shifting of the posterior dura [15]. Due to their occurrence after the peak age of the normal age curve, it is postulated that disproportionate growth between the vertebral column and spinal canal contents could be the underlying cause, and strenuous activity could be a precipitating factor [16,17].

Zhou et al. conducted a study on 192 patients with Hirayama disease in China and concluded that it is a benign, self-limiting disorder with juvenile preponderance and asymmetric distal atrophy of the distal upper arm with no geographic difference in clinical presentation [18].

Even though Hirayama disease is mainly seen in Asia, a few cases have been reported in the Western hemisphere as well. Increased awareness may lead to early diagnosis and intervention in North America [19-21].

Study limitations

Hirayama disease is a well-researched disease with accurate clinical and imaging criteria for the diagnosis. Through this study, we have comprehensively evaluated clinical presentations and tried to classify the severity of MRI findings for the Indian population. However, the possible shortcomings of this study are its relatively small sample size. Most of our cases were in the stage of chronic myelomalacia at the initial diagnosis. Moreover, it is a single-institute study and might not be representative of the national population.

## Conclusions

Hirayama disease is an uncommon type of cervical myelopathy seen typically in juvenile males. The occult onset of distal upper limb weakness and atrophy during puberty, typical MRI features of lower cervical cord atrophy, and the presence of a crescent-shaped enhancing mass in the posterior epidural space are pathognomonic of the condition. A few atypical cases can also occur, as described in our study. Early diagnosis and treatment are crucial to avoiding serious dysfunction.

## References

[1] Weerakkody Y, Sharma R, Eriksson B (2023). Radiopedia: Hirayama disease. https://doi.org/10.53347/rID-13903.

[2] Wang H, Tian Y, Wu J (2021). Update on the pathogenesis, clinical diagnosis, and treatment of Hirayama disease. Front Neurol.

[3] Raval M, Kumari R, Dung AA, Guglani B, Gupta N, Gupta R (2010). MRI findings in Hirayama disease. Indian J Radiol Imaging.

[4] Boruah DK, Prakash A, Gogoi BB, Yadav RR, Dhingani DD, Sarma B (2018). The importance of flexion MRI in Hirayama disease with special reference to laminodural space measurements. AJNR Am J Neuroradiol.

[5] Hirayama K, Tsubaki T, Toyokura Y, Okinaka S (1963). Juvenile muscular atrophy of unilateral upper extremity. Neurology.

[6] Hou C, Han H, Yang X (2012). How does the neck flexion affect the cervical MRI features of Hirayama disease?. Neurol Sci.

[7] Sonwalkar HA, Shah RS, Khan FK, Gupta AK, Bodhey NK, Vottath S, Purkayastha S (2008). Imaging features in Hirayama disease. Neurol India.

[8] Fujimori T, Tamura A, Miwa T, Iwasaki M, Oda T (2017). Severe cervical flexion myelopathy with long tract signs: a case report and a review of literature. Spinal Cord Ser Cases.

[9] Brandicourt P, Sol JC, Aldéa S, Bonneville F, Cintas P, Brauge D (2018). Cervical laminectomy and micro resection of the posterior venous plexus in Hirayama disease. Neurochirurgie.

[10] Chiba S, Yonekura K, Nonaka M, Imai T, Matumoto H, Wada T (2004). Advanced Hirayama disease with successful improvement of activities of daily living by operative reconstruction. Intern Med.

[11] Dohzono S, Toyoda H, Tamura A, Hayashi K, Terai H, Nakamura H (2019). Surgical treatment of a patient with prolonged exacerbation of Hirayama disease. Spine Surg Relat Res.

[12] Cortese R, Gerevini S, Dicuonzo F, Zoccolella S, Simone IL (2015). Hirayama disease: the importance of an early diagnosis. Neurol Sci.

[13] Wu W, Wang S, Lin J (2019). A 34-year-old female patient with Hirayama disease complicated by severe spinal cord injury. World Neurosurg.

[14] Lyu F, Zheng C, Wang H (2020). Establishment of a clinician-led guideline on the diagnosis and treatment of Hirayama disease using a modified Delphi technique. Clin Neurophysiol.

[15] Tashiro K, Kikuchi S, Itoyama Y (2006). Nationwide survey of juvenile muscular atrophy of distal upper extremity (Hirayama disease) in Japan. Amyotroph Lateral Scler.

[16] Huang YC, Ro LS, Chang HS, Chen CM, Wu YR, Lee JD, Lyu RK (2008). A clinical study of Hirayama disease in Taiwan. Muscle Nerve.

[17] Kohno M, Takahashi H, Yagishita A, Tanabe H (1998). "Disproportion theory" of the cervical spine and spinal cord in patients with juvenile cervical flexion myelopathy. A study comparing cervical magnetic resonance images with those of normal controls. Surg Neurol.

[18] Zhou B, Chen L, Fan D, Zhou D (2010). Clinical features of Hirayama disease in mainland China. Amyotroph Lateral Scler.

[19] Ghosh PS, Moodley M, Friedman NR, Rothner AD, Ghosh D (2011). Hirayama disease in children from North America. J Child Neurol.

[20] Dejobert M, Geffray A, Delpierre C, Chassande B, Larrieu E, Magni C (2013). Hirayama disease: three cases. Diagn Interv Imaging.

[21] Sim K, Gaillard F, Day T (2014). Anterior displacement of the spinal cord on flexion views in a patient with unilateral arm weakness. Hirayama's disease. J Clin Neurosci.

